# Clinical spectrum of *MTOR*-related hypomelanosis of Ito with neurodevelopmental abnormalities

**DOI:** 10.1038/s41436-021-01161-6

**Published:** 2021-04-08

**Authors:** Virginie Carmignac, Cyril Mignot, Emmanuelle Blanchard, Paul Kuentz, Marie-Hélène Aubriot-Lorton, Victoria E. R. Parker, Arthur Sorlin, Sylvie Fraitag, Jean-Benoît Courcet, Yannis Duffourd, Diana Rodriguez, Rachel G. Knox, Satyamaanasa Polubothu, Anne Boland, Robert Olaso, Marc Delepine, Véronique Darmency, Melissa Riachi, Chloé Quelin, Paul Rollier, Louise Goujon, Sarah Grotto, Yline Capri, Marie-Line Jacquemont, Sylvie Odent, Daniel Amram, Martin Chevarin, Catherine Vincent-Delorme, Benoît Catteau, Laurent Guibaud, Alexis Arzimanoglou, Malika Keddar, Catherine Sarret, Patrick Callier, Didier Bessis, David Geneviève, Jean-François Deleuze, Christel Thauvin, Robert K. Semple, Christophe Philippe, Jean-Baptiste Rivière, Veronica A. Kinsler, Laurence Faivre, Pierre Vabres

**Affiliations:** 1 INSERM UMR1231, Bourgogne Franche-Comté University, Dijon, France; 2 MAGEC-Mosaïque Reference Center, Dijon University Hospital, Dijon, France; 3 Neuropaediatrics and Development Pathology Department, Trousseau Hospital, AP–HP, Paris, France; 4 Genetics Department and Reference Center for rare causes of Intellectual Disability, Pitié-Salpêtrière hospital, AP-HP, Paris, France; 5 Plateforme IBiSA de Microscopie Electronique, Anatomie et cytologie pathologique, Université et CHRU de Tours, Tours, France; 6 INSERM U1259 MAVIVH, Université et CHRU de Tours, Tours, France; 7 Fédération Hospitalo-Universitaire Médecine Translationnelle et Anomalies du Développement (TRANSLAD), Dijon-Burgundy University Hospital, Dijon, France; 8 Pathology department, Dijon-Burgundy University Hospital, Dijon, France; 9 The University of Cambridge Metabolic Research Laboratories, Institute of Metabolic Science, Cambridge, UK; 10 Pediatrics and Medical Genetics Department, Dijon-Bourgogne University Hospital, Dijon, France; 11 Service d’Anatomie et Cytologie Pathologique, Necker-Enfants Malades Hospital, Paris, France; 12 Paediatric Dermatology, Great Ormond St Hospital for Children NHS Foundation Trust, London, UK; 13 UCL GOS Institute of Child Health, London, UK; 14 Mosaicism and Precision Medicine laboratory, Francis Crick Institute, London, UK; 15 National Genotyping Center, Genomic Institute, CEA, Evry, France; 16 Clinical Genetics department, Rennes University Hospital, Rennes, France; 17 Genetics Department, AP-HP, Robert-Debré University Hospital, Paris, France; 18 Medical Genetics Unit, CHU La Réunion, Saint-Pierre, France; 19 Clinical Genetics Department, Créteil Hospital, Créteil, France; 20 Unité Fonctionnelle Innovation en Diagnostic Génomique des Maladies Rares, FHU-TRANSLAD, CHU Dijon Bourgogne University Hospital, Dijon, France; 21 Medical Genetic Department, Jeanne de Flandre Hospital, Lille, France; 22 Dermatology department, Lille University Hospital, Lille, France; 23 Pediatric and Fetal Imaging Department, Hospices Civils de Lyon, Bron, France; 24 Department of Paediatric Clinical Epileptology, Sleep Disorders and Functional Neurology, University Hospitals of Lyon (HCL), Lyon, France; 25 Brain Dynamics and Cognition (DYCOG) Team, Lyon Neuroscience Research Centre, Lyon, France; 26 Cytogenetics Department, Dijon University Hospital, Dijon, France; 27 Medical genetics department, Pôle Femme et Enfant, Clermont-Ferrand University Hospital–Hôpital d’Estaing, Clermont-Ferrand, France; 28 Dermatology Department, Montpellier University Hospital, Montpellier, France; 29 Medical Genetics Department, Montpellier University Hospital, Montpellier, France; 30 Centre de Référence Déficiences Intellectuelles de Causes Rares, Hôpital d’Enfants, Dijon, France; 31 Center for Cardiovascular Science, University of Edinburgh, Edinburgh, UK; 32 Centre de Référence Anomalies du Développement et Syndromes Malformatifs, Hôpital d’Enfants, Dijon, France

## Abstract

**Purpose:**

Hypomelanosis of Ito (HI) is a skin marker of somatic mosaicism. Mosaic *MTOR* pathogenic variants have been reported in HI with brain overgrowth. We sought to delineate further the pigmentary skin phenotype and clinical spectrum of neurodevelopmental manifestations of *MTOR*-related HI.

**Methods:**

From two cohorts totaling 71 patients with pigmentary mosaicism, we identified 14 patients with Blaschko-linear and one with flag-like pigmentation abnormalities, psychomotor impairment or seizures, and a postzygotic *MTOR* variant in skin. Patient records, including brain magnetic resonance image (MRI) were reviewed. Immunostaining (*n* = 3) for melanocyte markers and ultrastructural studies (*n* = 2) were performed on skin biopsies.

**Results:**

*MTOR* variants were present in skin, but absent from blood in half of cases. In a patient (p.[Glu2419Lys] variant), phosphorylation of p70S6K was constitutively increased. In hypopigmented skin of two patients, we found a decrease in stage 4 melanosomes in melanocytes and keratinocytes. Most patients (80%) had macrocephaly or (hemi)megalencephaly on MRI.

**Conclusion:**

*MTOR*-related HI is a recognizable neurocutaneous phenotype of patterned dyspigmentation, epilepsy, intellectual deficiency, and brain overgrowth, and a distinct subtype of hypomelanosis related to somatic mosaicism. Hypopigmentation may be due to a defect in melanogenesis, through mTORC1 activation, similar to hypochromic patches in tuberous sclerosis complex.

**Graphical Abstract:**

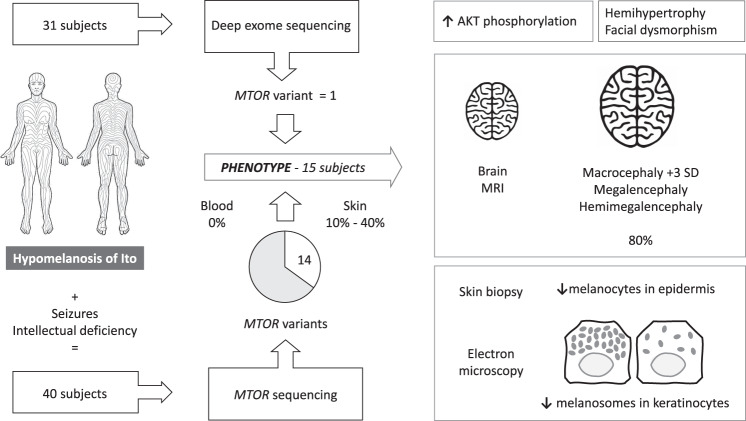

## INTRODUCTION

Hypopigmentation along Blaschko’s lines defines hypomelanosis of Ito (HI). Although in the initial description^1^ extracutaneous findings were not reported, HI was later recognized as a neurocutaneous disorder because of the frequency of brain involvement and epilepsy.^2–4^ Hemihypertrophy and additional developmental anomalies also appear to be more common in HI.^5,6^ In some patients, it may be difficult to determine whether the affected skin is hypopigmented or hyperpigmented,^7^ hence the name “pigmentary mosaicism,” encompassing both types of linear dyschromia.^8^ For hypopigmentation, the denominations “pigmentary mosaicism of the Ito type” or “linear hypomelanosis in narrow bands” have also been suggested.^9^

The genetic basis of HI is only partially understood. It has long been recognized as a hallmark of somatic mosaicism, since multiple nonrecurrent mosaic chromosomal anomalies have been reported in patients with this clinical feature.^10,11^ In contrast to chromosomal anomalies however, few mosaic single-gene anomalies have been reported in pigmentary mosaicism so far. In rare cases, mosaic activating *MTOR* pathogenic variants have been detected in the brain, blood, or buccal swabs (Supplementary Table 1), but in most patients, genetic testing was not performed on skin biopsy, and their functional consequences on skin pigmentation were not studied.

Besides somatic mosaic *MTOR* pathogenic variants in brain tissue, germline *MTOR* pathogenic variants have been reported in patients with Smith–Kingsmore syndrome (OMIM 616638) (Supplementary Figure 1), which consists of intellectual disability with macrocephaly, ventriculomegaly, seizures, and facial dysmorphism (midface hypoplasia, hypertelorism, downslanted palpebral fissures, depressed nasal bridge, thin upper lip, flat philtrum).

To characterize hypomelanosis of Ito with neurodevelopment anomalies related to *MTOR* variants clinically and genetically, we performed in-depth phenotyping in HI patients harboring mosaic *MTOR* pathogenic variants in the skin, including brain magnetic resonance imaging (MRI) and microscopic and ultrastructural skin analysis. We now provide further delineation of the cutaneous and neurodevelopmental spectrum associated with *MTOR* postzygotic pathogenic variants.

## MATERIALS AND METHODS

### Study subjects

Patients were ascertained by clinical geneticists or dermatologists in ten French centers (Dijon, Lille, Montpellier, Créteil, Paris-Trousseau, Rennes, Clermont-Ferrand, Reims, Paris-Robert-Debré, La Réunion), as part of a nationwide collaborative effort for identification of genes involved in cutaneous mosaic syndromes. Inclusion in the cohort required presence of both cutaneous and extracutaneous manifestations. Inclusion criteria in the phenotype study consisted of skin or hair hypopigmentation in a mosaic pattern—linear, segmental, or flag-like—associated with any type of extracutaneous involvement, and presence of a postzygotic *MTOR* variant. Criteria of noninclusion were diagnoses of Mendelian disorders of pigmentation, particularly Waardenburg syndrome and piebaldism, or hypochromic bands resulting from linear inflammatory conditions, such as lichen striatus or inflammatory linear verrucous epidermal nevus (ILVEN). All clinical features were recorded on a clinical research form, and cutaneous images were centralized and reviewed at the coordination center by two experts (A.S., P.V.). Cerebral MRI was performed at each center and centralized reading was performed by three other experts (C.M., D.R., L.G.).

### Next-generation sequencing

DNA analysis was performed at Dijon GAD laboratory, Université de Bourgogne, for 13 patients, and at University College London for 2 patients. Genomic DNA was extracted from blood, 5-mm punch biopsies of hypopigmented skin, cultured skin fibroblasts, buccal swabs, and saliva specimens. Details on next-generation sequencing are available in Supplementary Methods.

In-depth exome sequencing on whole-skin biopsies and blood genomic DNA was initially performed in 31 patients with hypopigmented bands along Blaschko’s lines or segmental hypochromic patches, associated with various extracutaneous features (flow diagram in Supplementary Figure 2). We found a mosaic *MTOR* pathogenic variant in the skin from one patient with hemimegalencephaly (HMEG) and severe epilepsy. Other patients carried a mosaic chromosomal abnormality (7 cases), a germline X-linked variant in *TFE3*^12,13^ (1 case), a postzygotic variant in *RHOA*^14^ (2 cases) or other candidate genes (4 cases), or remained negative (16 cases). Targeted deep sequencing of *MTOR* was subsequently performed on hypopigmented skin and/or blood DNA from 40 additional patients with Blaschko-linear hypopigmentation associated with epilepsy or intellectual deficiency who were subsequently ascertained (Supplementary Figure 2).

### Assessment of mTOR activation on cultured skin fibroblasts

Fibroblasts were obtained from a skin biopsy of patients P03 and P12. The presence of c.4556C>T (p.[Ala1519Val]) and c.7255G>A (p.[Glu2419Lys]) substitutions was checked both by Sanger sequencing (ABI BigDye Terminator Cycle Sequencing kit [v.3.1] and an ABI 3130 Genetic Analyzer, Applied Biosystems, Villebon-sur-Yvette, France), and TUDS, which allowed determination of variant allele fractions (VAFs). A mixed culture of nonmutant and *MTOR* mutant skin fibroblasts (p.[Glu2419Lys], VAF = 40%), three wild-type control fibroblast cultures (C1, C2, C3), and mixed cultures of nonmutant and *PIK3CA-*mutant control fibroblasts (p.[Gly1049Arg], VAF = 40%), M098 (p.[Gly418Lys], VAF = 32%), M018 (p.[Gln546Lys], VAF = 40%), and M032 (p.[His1047Arg], VAF = 30%) were studied. Cells were maintained at 37 °C in a humidified incubator in DMEM supplemented with 10% FBS, 1,000 u/l penicillin, 0.1 g/l streptomycin, and 2 mmol/l L-glutamine. Amino acid deprivation procedures were conducted as previously described.^15,16^

Phosphorylation of AKT at residue 473 was assessed using a direct enzyme-linked immunosorbent assay (ELISA) kit (InstantOne®, eBioscience, Cambridge, UK, #85-86042-253). Results were expressed relative to control levels and pooled; statistical analysis was performed using one-way analysis of variance (ANOVA) with Tukey’s post hoc analyses. Phosphorylation of p70S6K at residue 389 was assessed by immunoblotting and antibody labeling by Calnexin (#2679) and p70S6Kthr389 (#9234, Cell Signalling Technology, London, UK). Cell size was measured on 10,000 cells per run on an MS3 multisizer (Beckmann-Coulter), after incubation of fibroblasts with or without amino acids, since phosphorylation of the PI3K-AKT-MTOR pathway depends on nutrients.

### Microscopy

Additional skin biopsies for optical and electron microscopy were obtained from patients in each center and standard FFPE skin sections were processed prior to centralized analysis. Immunoperoxidase staining was performed on FFPE sections from P12 (hypopigmented and normal skin), P05, and P06 (hypopigmented skin only) and two control FFPE sections from age-matched controls, using anti-MITF (#NCL-L-MITF, 1:500, Leica, Nanterre, France) and anti-Melan-A (1:200; ab731; Abcam, Cambridge, MA, USA) mouse monoclonal antibodies. Glutaraldehyde-fixed skin biopsies from P12 and P11 were prepared for electron microscopy following standard procedures. Ultrathin sections (120 nm thick) were observed on a JEOL JEM-1011 transmission electron microscope (JEOL, Croissy, France) operating at 100 kV and pictures taken using ES1000W Erlangshen CCD camera (GATAN, Elancourt, France). Stage I and stage IV melanosomes were counted in 15 melanocytes from biopsies taken from both normal (control) and hypomelanotic skin from patients P12 and P11, respectively. Melanosomes were counted in 50 basal layer keratinocytes for each sample. Statistical significance of differences was assessed using Wilcoxon rank sum test, with a *p* value < 0.05 considered significant.

## RESULTS

**Fig. 1:**
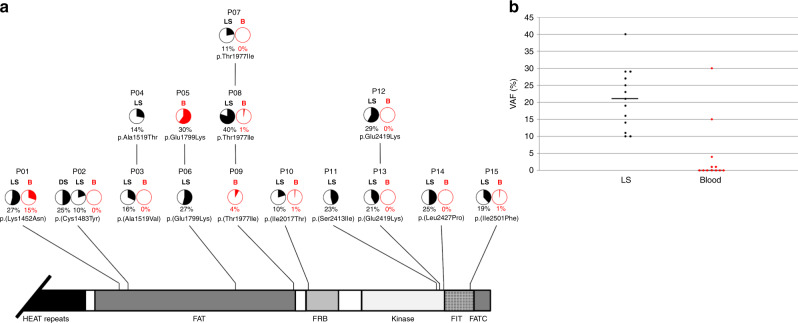
Distribution of postzygotic missense *MTOR* variations. (**a**) Structure of mTOR protein including the Huntingtin, Elongation factor 3, protein phosphatase 2A, and TOR1 (HEAT) repeat; FAT (FRAP, ATM, and TRRAP) domain; FRB (FKBP12-rapamycin binding) domain; kinase (serine-threonine kinase kinase) domain; FATC (FAT, FRAP, ATM and TRRAP carboxy-terminal) domain; and FIT (Found in TOR) domain. Variant allele fractions (VAFs) (%) are shown as pie charts with a maximum allele fraction of 50%. B (red) blood, DS dark skin, LS light skin. Variation details are summarized in Supplementary Table 3. (**b**) Distribution of VAFs in hypopigmented skin (LS, black dots) and blood (red dots), with median values.

In one affected individual from the initial cohort of 31 patients (P12), exome sequencing on DNA from hypopigmented skin identified a de novo predicted missense p.(Glu2419Lys) (c.7255G>A) *MTOR* variant, present in 21 of 74 reads (28%), absent from blood-derived DNA from her unaffected parents (Supplementary Table 2). The variant was absent from the gnomAD variant database, found to affect a highly conserved nucleotide and amino acid, and was predicted as likely deleterious in silico. TUDS of the *MTOR* kinase domain harboring the c.7255G>A substitution confirmed its presence in 29% of alleles in whole skin biopsy, and 41% of alleles in cultured skin fibroblasts. The variant was nearly undetectable in blood (less than 1% of reads) (Fig. 1).

In the second cohort of 40 patients, this variation and ten additional postzygotic *MTOR* substitutions were identified in 14 unrelated patients (35.0%): p.(Lys1452Asn), p.(Cys1483Tyr), p.(Ala1519Val), p.(Ala1519Thr), p.(Glu1799Lys), p.(Thr1977Ile), p.(Ile2017Thr), p.(Ser2413Ile), p.(Leu2427Pro), and p.(Ile2501Phe). Hypomelanotic skin samples were available for further study in 13/15 individuals with mosaic *MTOR* variants, and normally pigmented skin DNA was also available in one patient (P02). Skin VAFs varied between 10% and 40% (median = 21%). Blood samples were available in 12/15 individuals. *MTOR* variants were found in blood in 6/12 patients, with lower VAFs than in the skin, between 1% and 30% (median = 0%) (Fig. 1, Supplementary Table 3). Recurrent *MTOR* hotspots were identified in nine patients: p.(Glu1799Lys), p.(Glu2419Lys), and p.(Ala1519Thr)/p.(Ala1519Val) in two patients, as well as p.(Thr1977Ile) in three patients. Overall, five variants (p.[Lys1452Asn], p.[Ala1519Thr], p.[Ala1519Val], p.[Ile2017Thr], p.[Ser2413Ile]) had never been reported previously.

Both p.(Glu2419Lys) and p.(Ile2017Thr) *MTOR* variants were previously found to result in constitutive mTOR complex 1 (mTORC1) activity in vitro and in vivo.^17,18^ Primary skin fibroblasts from P12, with a VAF of 40%, were used to test p.(Glu2419Lys] mTOR activity by assessing AKT and p70 ribosomal protein S6 kinase (p70S6K) phosphorylation status^15,19^ (Supplementary Figure 3). ELISA (Supplementary Figure 3a) and immunoblotting experiments (Supplementary Figure 3b) following amino acid deprivation showed increased levels of phosphorylated AKT^ser473^ and p70S6K^thr389^ in mutant cells compared with controls. AKT^ser473^ and p70S6K^thr389^ phosphorylation levels were similar to those of skin fibroblasts harboring *PIK3CA* activating variants (Supplementary Figure 3). However, in amino acid–rich conditions, the p.(Glu2419Lys) *MTOR* mutant cells showed no constitutive activity, in contrast with *PIK3CA* mutant cell lines (Supplementary Figure 3c). We found no significant differences in median cell diameter between mutant and control cells (Supplementary Figure 3d). In comparison, in primary cultured fibroblasts harboring the p.(Ala1519Val) *MTOR* variant at very low levels (<1%), phosphorylation was not increased at baseline or under amino acid deprivation.

**Fig. 2:**
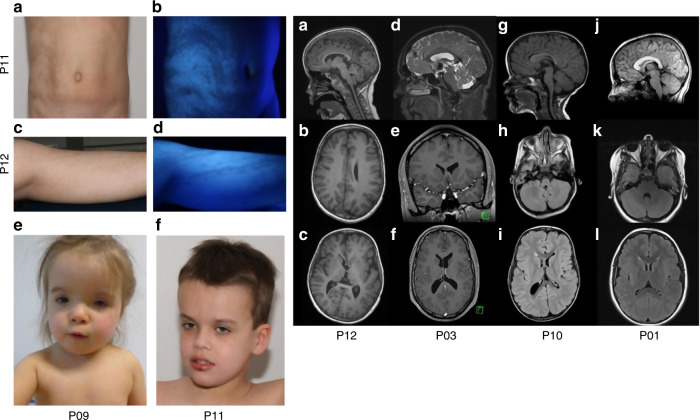
Clinical pigmentary skin phenotype and brain imaging. Clinical pigmentary skin phenotype in two patients (Left). (**a**, **b**) P11. (**a**) Unilateral linear and whorled hypopigmentation in multiple large bands with ragged border and sharp midline limitation on the abdomen. (**b**) Enhanced contrast on Wood’s lamp illumination. (**c**, **d**) P12. (**c**) Linear hypopigmentation in large bands on left lower limb. (**d**) Enhanced contrast on Wood’s lamp illumination. (**e**, **f**) Facial features of patients P09 and P11. Brain magnetic resonance image (MRI) of subjects P01, P03, P10 and P12 (Right). (**a**–**c**) P12. Left HMEG with altered ventricle shape (small frontal horn), enlarged left thalamus and caudate nucleus (**a**), slightly thickened cortical mantle and enlarged white matter with normal signal (**b**) and enlarged anterior corpus callosum (**c**). (**d**–**f**) P03. Normal corpus callosum (**d**), small frontal horns (**e**, **f**), and mild overgrowth of the right cerebral hemisphere, with slight enlargement of the right posterior ventricle (**c**). (**g**–**i**) P10. Normal corpus callosum on sagittal section (**g**), overgrowth of the right cerebellar hemisphere (**h**), right posterior ventricle enlargement, small frontal horn, increased white matter volume and thickened cortical mantle (**i**). (**j**–**l**) P01. Thickened corpus callosum (**j**), with symmetrical enlargement of cerebellar (**k**) and cerebral hemispheres (**l**).

**Fig. 3:**
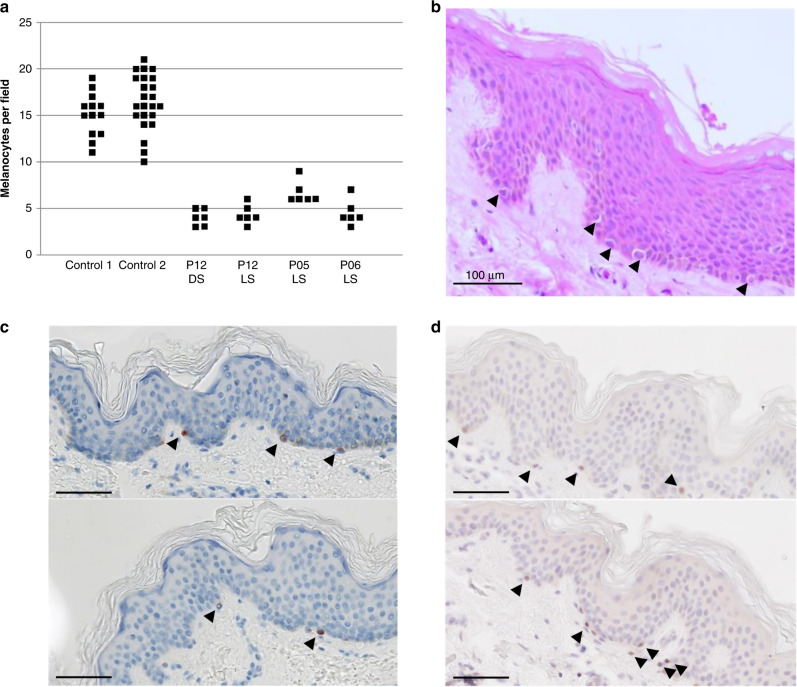
Microscopy of paraffin embedded skin biopsies. (**a**) Numbers of melanocytes per field on whole skin sections from patients P05, P06, and P12 (patients: 6 fields; controls: 13 and 24 fields; hematoxylin eosin [HE] staining). DS dark skin, LS light skin. (**b**) HE staining from P06 hypomelanotic skin. (**c**) Subject P12. Melan-A immunohistochemistry on skin biopsy sections from pigmented skin (top) and hypomelanotic skin (bottom). (**d**) Subject P12. MITF labeling on biopsy sections from pigmented skin (top) and hypomelanotic skin (bottom). Scale bar: 100 µm. Melanocytes are shown by arrowheads.

The 15 patients (10 females and 5 males) aged 1–30 years (median: 6 years), consisted of 13 children aged 14 or younger and two adults. Fourteen patients had either well-limited or ill-defined linear hypopigmentation along Blaschko’s lines, in a typical S-shaped, V-shaped or whorled pattern, more visible under Wood’s light, mainly located on the trunk and lower limbs, without lateral predominance (Fig. 2, Supplementary Table 4). One additional patient did not have linear hypomelanosis, but instead a hypopigmented patch of scalp hair, a segmental cutaneous flag-like hypopigmented patch on his shoulder, and iris heterochromia. Woolly/curly hair was found in four patients, but no other anomalies of the integument or mucous membranes were noted. Since a decrease in melanocyte numbers and melanosome maturation has been reported in ash-leaf macules of tuberous sclerosis patients harboring loss-of-function variants in *TSC1* or *TSC2*, which encode upstream inhibitors of mTOR, we studied melanogenesis in patients with *MTOR*-related HI. Biopsies from hypomelanotic and normally pigmented skin in patients P05, P06, P11, and P12, showed fewer normal melanocytes per standardized field on Melan-A immunostaining compared with controls (mean = 15.8 melanocytes/field for control skins and mean = 5.2 melanocytes/field in HI skins, *p* = 2.6.10^−9^) (Fig. 3). On hypomelanotic skin biopsies, MITF expression appeared normal, since anti-MITF labeling in epidermal melanocytes was unchanged (Fig. 3). MITF labeling was either positive (P12) or absent (P05 and P06). We failed to identify downregulation of MITF in primary skin fibroblasts from one patient (data not shown).

**Fig. 4:**
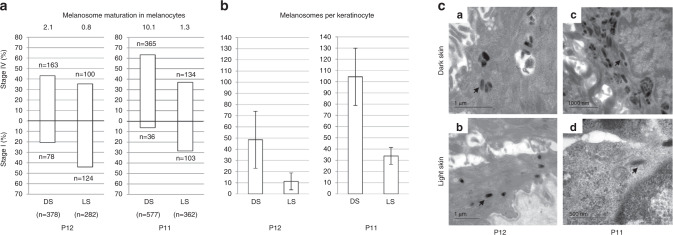
Ultrastructural study. (**a**) Melanosome maturation in melanocytes: Melanosome count in melanocytes on skin biopsy sections from pigmented (black) and hypopigmented area (white) in patients P12 (left) and P11 (right). Melanosomes were counted in 15 melanocytes. Upper bars: stage I melanosome. Lower bars: stage IV melanosomes (numbers at top of each bar). Ratio and total number of counted melanosomes are given at top and bottom of each figure. (**b**) Melanosome quantification in keratinocytes. Mean number of melanosomes per basal layer keratinocyte (*n* = 50) (**c**). Transmission electron microscopy (TEM) of melanocytes at the basal epidermal layer in subjects P12 (**a**, **b**) and P11 (**c**, **d**). Melanosomes (arrows), in dark (**a**–**c**) and hypopigmented (**b**–**d**) areas. DS dark skin, LS light skin.

In patients P12 and P11, the number of stage IV melanosomes were markedly decreased in 15 melanocytes from hypomelanotic skin (*n* = 378 and *n* = 282, respectively), compared with 15 melanocytes from normal skin (*n* = 577 and *n* = 362, respectively) (*p* = 0.03 for P12 and *p* = 0.0002 for P11) (Fig. 4). Likewise, the stage IV/stage I melanosome ratio in melanocytes from hypomelanotic skin was reduced by 61.9% compared with normal skin in patient P12, and by 87.3% in patient P11. Mean numbers of melanosomes per cell were also decreased in keratinocytes from hypomelanotic skin as compared with normal skin: by 75.0% in patient P12, and by 67.6% in patient P11 (*p* value = 8.2.10^−16^ and 3.8.10^−15^ for P12 and P11).

**Fig. 5:**
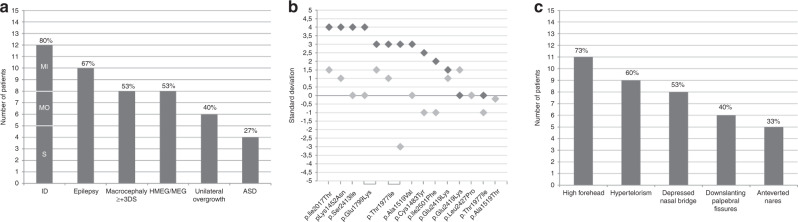
Clinical features in hypomelanosis of Ito (HI) individuals with *MTOR* postzygotic pathogenic variants. (**a**) Frequencies of neurological involvement and overgrowth. (**b**) Standard deviations for occipitofrontal circumference (OFC) (dark gray) and body height (light gray). (**c**) Frequencies of recurrent facial features. Percentages were calculated with the number of patients with available data as the denominator. ID intellectual disability, MI mild, MO moderate, S severe.

Twelve patients had psychomotor impairment ranging from mild (first steps achieved at 20 months) to severe (sitting position achieved at 6 years). All of them subsequently had intellectual disability (ID). Autism spectrum disorder was present in four patients, who also had ID. Eight patients had macrocephaly (occipitofrontal circumference [OFC] ≥ +3 SD), associated with unilateral body overgrowth in four of them. One patient had slightly elevated OFC (+2.5 SD). Six patients had normal OFC, one of them with hemihypertrophy, (Fig. 5, Supplementary Table 4). Seizures were diagnosed in ten patients, including two without ID (Fig. 5, Supplementary Table 4). Among patients with seizures, only five had macrocephaly, whereas all five patients without epilepsy had macrocephaly.

Brain MRI was performed in ten patients: three with normal OFC and seven with macrocephaly. Among patients with macrocephaly, MRI showed normal symmetric brains without megalencephaly (MEG) (normal lateral ventricles and pericerebral spaces) in four (Supplementary Table 4, Fig. 2). Among the ten patients who had seizures, MRI was performed in six and showed HMEG in all of them, either with normal OFC (<+3 SD, in three patients) or macrocephaly (OFC between +3 and +4 SD, in the three others). All six cases of HMEG were characterized on MRI by (1) increased volume of the white matter; (2) absence of signal change, except in one case with bilateral frontal gray matter heterotopia; and (3) cortex thickening in two patients. HMEG was also associated with homolateral (four patients) or bilateral (one patient) narrowing of lateral ventricle frontal horns, homolateral enlargement of the thalamus and caudate nucleus (one patient), or homolateral enlargement of cerebellum hemispheres (four patients). In the five patients without epilepsy, MRI was performed in four and showed either (2 cases) or normal brain (2 cases). Overall, 12 patients in our series (80%) had either macrocephaly or HMEG/MEG.

Facial dysmorphism (Fig. 5, Supplementary Table 4) consisted of hypertelorism, prominent forehead, depressed nasal bridge, or downslanting palpebral fissures. In addition to iris heterochromia in two cases, ocular involvement consisted of strabismus/amblyopia, astigmatism, myopia, hypermetropia, coloboma, or retinitis pigmentosa. Renal anomalies usually found in tuberous sclerosis complex (TSC) (angiomyolipomas and renal cysts) were absent in patients with mosaic *MTOR* pathogenic variants. On ultrasonography, 2/12 patients had increased renal cortex echogenicity, a nonspecific finding.

## DISCUSSION

In this group of 71 patients with patterned dyspigmentation, we identified a subset due to mosaicism for MTOR pathogenic variants. We have further delineated the clinical spectrum of *MTOR*-related HI with neurodevelopmental anomalies (Supplementary Table 4). These features include ID, macrocephaly, HMEG, and epilepsy, in addition to a pigmentation pattern suggestive of mosaicism. This combination of neurocutaneous features has previously been reported (Supplementary Table 5) in seven patients with postzygotic *MTOR* activating pathogenic variants (Supplementary Table 1). Two reported siblings with a germline *MTOR* activating variant had a pigmentary phenotype without further description.^20^

The severity of neurodevelopmental phenotypes varied greatly among our patients. Intellectual functioning ranged from normal to severe ID. Patients without ID had epilepsy or MEG. This neurodevelopmental spectrum overlaps with Smith–Kingsmore syndrome (SKS), caused either by germline *MTOR* pathogenic variants, or mosaic *MTOR* pathogenic variants with high VAFs in the brain.^20^ Presence of HI, absent in SKS patients with a constitutional heterozygous *MTOR* pathogenic variant, may be an indication of mosaicism and the need for further studies. To date, ID has been reported in all patients with SKS or postzygotic *MTOR* pathogenic variants and HMEG. Severe ID was present in patient P15, who harbored a postzygotic p.(Ile2501Phe) change. In contrast, two individuals from one previously published family, who carried a germline p.(Ile2501Val) variant, had normal cognition, focal epilepsy, and normal OFC.^21^ Since discrepancies in the cerebral phenotype for an identical germline variant cannot be explained by different amounts of mutant brain cells, the very mild phenotype in this family would be better explained by a milder gain-of-function effect of the p.(Ile2501Val) substitution than of the p.(Ile2501Phe) substitution that we found in a mosaic state in our patient.

MRI showed HMEG in all six cases with seizures, whereas in four patients without epilepsy, MRI showed either MEG or a normal brain. This suggests that, in patients with postzygotic *MTOR* pathogenic variants, HMEG might be more epileptogenic than MEG. MRI did not show cortical or other cerebral malformations in the two MEG patients and in three of the HMEG patients, while only three patients with HMEG had focal cortex thickening suggestive of polymicrogyria or periventricular heterotopia. Hence, our series also supports that, in patients with a neurodevelopmental phenotype, HMEG or MEG without apparent brain malformation might be more suggestive of pathogenic variants in *MTOR* rather than in *PIK3CA* or *AKT3*, as previously outlined.^22–25^ Interestingly, the structure and function of homogeneously enlarged brains with postzygotic *MTOR* pathogenic variants may be less altered than of those with HMEG. Indeed, previous studies comparing phenotypes related to either postzygotic or germline *MTOR* pathogenic variants have suggested that severity of neurodevelopmental impairment does not correlate with the proportion of affected brain cells.^20,21,25^ This is likely explained by increased strength of the gain-of-function effect of mosaic pathogenic variants, which would result in embryonic lethality in the germline state, compared with a milder effect of germline pathogenic variants, which would be compatible with fetus survival.

A pigmentary phenotype with hypopigmentation and hyperpigmentation has previously been reported in one of two sibs who both harbored a germline p.(Phe2202Cys) *MTOR* variant,^20^ but its precise clinical description is missing, and no conclusions can be drawn from this case about the role of germline *MTOR* pathogenic variants on skin pigmentation. All patients in our series carried postzygotic *MTOR* pathogenic variants in their skin, which were absent from blood in 50% of cases tested. Linear hypopigmentation has been considered a nonspecific manifestation of mosaicism, as various types of nonrecurrent chromosome anomalies were initially reported in association with the phenotype, and some authors have even suggested that the term “hypomelanosis of Ito” should be abandoned.^11^ However, we and others have now found mosaic single-gene defects in HI, such as *MTOR* or *RHOA* pathogenic variants.^14^ In one case of the phenotypic “opposite” of HI, linear and whorled nevoid hypermelanosis,^26^ where Blaschko-linear hyperpigmentation is the cardinal cutaneous feature, we have also described a pathogenic mosaic variant in the *KITLG* gene.^27^ Hence, hypo/hyperpigmentation along Blaschko’s lines should no longer be considered merely as a clinical marker of somatic mosaicism, since, in combination with extracutaneous findings, it may also specifically point at the causative gene.

The *MTOR* gene encodes the mechanistic target of rapamycin (mTOR). This serine-threonine kinase is highly conserved, and is an essential component of MTORC1 and MTORC2 complexes. The PI3K-AKT-mTOR signaling pathway is pivotal in cell growth, protein synthesis, autophagy, and cytoskeletal dynamics.^28–30^ Although MITF labeling studies were inconclusive, cutaneous hypopigmentation may directly result from mTOR complex hyperactivation. In TSC, a condition resulting from loss of function of *TSC1* or *TSC2*,^31^ activation of mTOR has been shown to suppress melanogenesis via MITF downregulation, resulting in typical ash-leaf or confetti-like hypopigmented macules.^32–34^ Repigmentation can be obtained by treatment with topical mTOR inhibitor rapamycin,^35^ since mTORC1 inhibition results in induction of melanogenesis through upregulation of MITF and its downstream targets TRP1 and TRP2.^32,33,36,37^ In hypopigmented skin of four patients with *MTOR*-related HI, we found a decrease in melanocytes, a defect in the maturation process of melanosomes, and a reduced number of melanosomes in keratinocytes, as well as activating *MTOR* variants and increased AKT and p70S6K phosphorylation in dermal fibroblasts from patients. Our findings are consistent with upregulation of mTORC1 resulting in partial suppression of melanogenesis. Paradoxically though, in P02, normally pigmented skin harbored a VAF of 25%, whereas the VAF was only 10% in hypopigmented skin. We hypothesize that lower VAFs may result from death of mutant cells in hypopigmented areas through autophagy, as recently shown in TSC.^33^ A decreased number of melanocytes was found both in hypopigmented and normal skin in P12 (Fig. 3), which supports this hypothesis.

Our results highlight the diagnostic importance of accurate clinical phenotyping of mosaic syndromes, and provide further evidence that genetic defects underlying HI extend beyond chromosomal mosaicism. Additional genetic defects remain to be identified in a number of patients with linear hypopigmentation and various associated manifestations. Yet, identification of mosaic *MTOR* pathogenic variants in HI with neurodevelopmental defects defines a clinically and genetically recognizable condition. It has direct clinical implications for genetic tests and genetic counseling.

Most *MTOR* pathogenic variants, not found in the germline state, are thought to be embryonic lethal. Hence, the risk of transmission from a mosaic patient harboring this type of variant appears low or absent. However, transmission of nonlethal pathogenic variants, resulting in offspring with SKS, is still possible. Although the positive predictive value of HI with neurodevelopmental anomalies for the diagnosis of *MTOR* somatic mosaicism is not yet known, in our series, *MTOR* pathogenic variants were found in more than one third (36.6%) of patients with HI and seizures. Likewise, macrocephaly and/or HMEG/MEG, found in 80% of cases in our series, appear as fairly specific, as they were absent from patients with mosaic *RHOA* pathogenic variants.^14,38^ Hence, in our opinion, such typical clinical presentation warrants *MTOR* sequencing by ultrasensitive methods. DNA from the skin or other affected tissue is required for genetic testing, since the VAF is higher in skin (21%) compared with blood (0%), where *MTOR* pathogenic variants are usually absent or at very low levels, close to next-generation sequencing detection limit (1% at a depth of 1,000×). Last, clinical consequences of mTORC1 upregulation, particularly neurological involvement, may be amenable to tailored treatment with mTOR inhibitors, although data on efficacy are inconclusive so far.^39^

## Data Availability

All *MTOR* pathogenic variants identified were submitted to the ClinVar database under the number SUB8228199 and data are available at https://www.ncbi.nlm.nih.gov/clinvar/?term=SUB8228199.

## Suplementary information

Supplementary material
