# Supplementary material for: Clinical spectrum of MTOR-related hypomelanosis of Ito with neurodevelopmental abnormalities
Source: Genet Med. 2021 Apr 8;23(8):1484–91. doi: 10.1038/s41436-021-01161-6 (PMC8354853; doi:10.1038/s41436-021-01161-6)
Supplement: Supplementary file 1 — Supplementary material [file 41436_2021_1161_MOESM1_ESM.pdf]

**SUPPLEMENTARY MATERIAL**

**Supplementary Method**

DNA Analysis

Exome capture and sequencing were performed at the Centre National de Génotypage (CNG, Evry, France) from 3 µg of genomic DNA for each patient sample. As a first approach, we performed deep exome sequencing on skin-derived and blood-derived DNA from 31 patients with pigmentary skin mosaicism, and blood from their parents. In subject P12, exome sequencing was performed on DNA obtained from a hypopigmented skin band (mean depth 200X), and blood-derived DNA from her unaffected parents (mean depth 80X). Data were processed as previously described [1]. Variant locations are based on the human genome reference sequence GRCh37/hg19. The Genome Analysis Toolkit (GATK) v.2.6-4 was used for base quality score recalibration, indel realignment, and variant discovery [2]. Candidate *de novo* events were systematically identified by focusing on protein-altering and splice-site variants. We assessed the presence of the identified variants in public variant databases, namely the Genome Aggregation Database (gnomAD, http://gnomad.broadinstitute.org/) and the Catalogue of somatic variants in cancer (COSMIC, http://cancer.sanger.ac.uk/cancergenome/projects/cosmic/).

Targeted deep sequencing was performed on all 57 coding regions of *MTOR* (reference accession LRG_734_t1). Sequences were amplified using custom intronic primers (sequences available on demand) and standard long-range PCR protocols. Libraries were prepared using the transposase-based Nextera XT DNA Sample Preparation kit (Illumina, Evry, France), and sequenced on a MiSeq instrument (Illumina, Evry, France) according to the manufacturer's recommendations.

Nucleotide-level conservation and impact of amino acid substitutions were assessed using Genomic Evolutionary Rate Profiling (GERP), Polyphen-2 (HumVar-trained model), and Combined Annotation-Dependent Depletion (CADD) scores. All prediction scores are listed in Supplementary Table 3.

Exome and targeted deep sequencing were performed according to standard protocol (detailed in Supplementary data). All MTOR variants identified were submitted to the CLINVAR database under

the number SUB8228199.

**Supplementary Table 1.** Previously reported *MTOR* variants in affected individuals with pigmentary features.

| ***MTOR* variant**<br>**Amino acid change** | **Type of variant** | **Amino acid change*** | **Available clinical features** | **References** |
|---|---|---|---|---|
| chr1:g.11217231A>G | Somatic | p.(Cys1483Arg) | Hemimegalencephaly, **hypomelanosis of Ito** | Lee JH *et al* 2012 [3] |
| | Somatic | | Macrocephaly, ID, facial dysmorphism, **linear hyperpigmentation** | Gordo G *et al*, 2018 [4] |
| chr1:g.11188164G>T | Somatic | p.(Thr1977Lys) | Three unrelated patients with diffuse asymmetric MEG and **hypomelanosis of Ito** | Mirzaa GM *et al*, 2016 [5] |
| | Somatic | | One patient with MEG, asymmetric polymicrogyria, hypotonia, and **hyperpigmentation** | Handoko M *et al*, 2019 [6] |
| chr1:g.11184612T>G | Germline | p.(Phe2202Cys) | Two siblings with macrocephaly, severe ID, capillary malformations (face and shoulder) and **area of hypo/hyperpigmentation** | Gordo G *et al*, 2018 [4] |
| chr1:g.11184573G>A | Somatic | p.(Ser2215Phe) | One patient with HMEG, severe ID, seizures, **hypochromic patches** | Pelorosso C *et al*, 2019 [7] |

*Variant locations are based on GRCh37/hg19 and reference *MTOR* accession LRG_734_t1.

MEG: megalencephaly; FCD: Focal Cortical Dysplasia; ID: Intellectual Disability; HMEG::hemimegalencephaly.

**Supplementary Table 2.** Summary of exome sequencing experiments in subject PED1004 and her unaffected parents

| Individual | DNA source | Target size (Mb)[a] | Aligned bases (Gb)[b] | Mean sequencing depth[c] | Percent target ≥ 10x[c] | Percent target ≥ 100x[c] |
|---|---|---|---|---|---|---|
| PED1004 | Skin biopsy | 51 | 20.798 | 242 | 96.5 | 81.6 |
| Father | Blood | 51 | 9.498 | 130 | 96.0 | 56.5 |
| Mother | Blood | 51 | 7.035 | 91 | 95.2 | 37.5 |

Mb, megabases; Gb, gigabases. [a]Target size of the SureSelect Human All Exon V5 kit (Agilent). [b]Bases from "P*assing Filter" (PF) reads* mapped to the human genome reference sequence (GRCh37/hg19 build of UCSC Genome Browser, see http://genome.ucsc.edu/). [c]Sequencing depth metrics were calculated using RefSeq coding exons and splice junctions as targets. Only reads with mapping quality ≥ 20 and bases with base quality ≥ 20 were considered.

**Supplementary Table 3.** Summary of mosaic *MTOR* changes

| Subject | *MTOR* change | COSMIC ID | Gnomad allele frequency | GERP | Polyphen-2 | CADD |
|---|---|---|---|---|---|---|
| P01 | chr1:g.11217322T>A c.4356A>T p.(Lys1452Asn) | COSM462620 | 0/246,180 | 4.66 | 0.95 | 18.85 |
| P02 | chr1:g.11217230C>T c.4448G>A p.(Cys1483Tyr) | COSM462615 | 0/246,180 | 5.32 | 0.99 | 26.10 |
| P03 | chr1:g.11210197G>A c.4556C>T p.(Ala1519Thr) | COSM462614 | 0/246,138 | 5.16 | 0.98 | 34.00 |
| P04 | chr1:g.11210198C>T c.4555G>A p.(Ala1519Val) | COSM462614 | 0/246,138 | 5.16 | 0.997 | 22.5 |
| P05 P06 | chr1:g.11190804C>T c.5395G>A p.(Glu1799Lys) | COSM180789 | 0/246,180 | 5.54 | 0.878 | 26.3 |
| P07 P08 P09 | chr1:g.11188164G>A c.5930C>T p.(Thr1977Ile) | COSM6241477 | 0/246,096 | 3.73 | 0.96 | 9.68 |
| P10 | chr1:g.11187847A>G c.6050T>C p.(Ile2017Thr) | - | 0/246,180 | 5.80 | 0.99 | 24.00 |
| P11 | chr1:g.11174437C>A c.7238G>T p.(Ser2413Ile) | COSM4703642 | 0/246,180 | 5.89 | 1.00 | 32.00 |
| P12 P13 | chr1:g.11174420C>T c.7255G>A p.(Glu2419Lys) | COSM4187184 | 0/246,028 | 5.89 | 1.00 | 36.00 |
| P14 | chr1:g.11174395A>G c.7280T>C p.(Leu2427Pro) | COSM5044474 | 0/246,180 | 5.89 | 1.00 | 26.00 |
| P15 | chr1:g.11169374T>A c.7501A>T p.(Ile2501Phe) | COSM4140746 | 0/246,180 | 5.82 | 0.99 | 21.60 |

CADD, Combined Annotation-Dependent Depletion; COSMIC, Catalogue of somatic mutations in cancer; Gnomad: Genome Aggregation Database; GERP, Genomic Evolutionary Rate Profiling [20–22].

Presence of identified *MTOR* variants was assessed in several public variant databases, including dbSNP build 141, Gnomad Browser, and COSMIC. All variants were absent from dbSNP build 14, and Gnomad database. Variant locations are based the human genome reference sequence GRCh37/hg19 and reference *MTOR* accession is LRG_734_t1.

**Supplementary Table 4 : Phenotype and genotype** of the fifteen affected individuals with *MTOR* variants.

| **Patient ID** | | **P01** | **P02** | **P03** | **P04** | **P05** | **P06** | **P07** | **P08** | **P09** | **P10** | **P11** | **P12** | **P13** | **P14** | **P15** |
|---|---|---|---|---|---|---|---|---|---|---|---|---|---|---|---|---|
| Age* (Y) | | 12 | 3 | 27 | 7 | 3 | 6 | 9 | 4 | 2.5 | 2.5 | 7 | 14 | 5.5 | 1 | 30 |
| Sex | | F | M | M | M | F | F | F | F | F | M | M | F | F | F | F |
| Hypomelanosis | | Linear<br>Trunk (P) (R), lower limbs | Linear<br>R side | Linear<br>Upper limb (L) | Linear<br>Lower limb (L + R) | Linear<br>Diffuse (trunk, upper + lower limbs) | Linear<br>Diffuse (upper + lower limbs), trunk | Linear<br>Lower limbs (R) | Linear<br>Trunk (P), upper + lower limbs (L + R) | Linear<br>Trunk, (P), limbs | Flag-like<br>Upper limb (L), scalp hair patch (R) | Linear<br>Trunk, back, groin (R) | Linear<br>Trunk (A), Lower +, upper limbs (L) | Linear<br>Trunk (R) | Linear<br>Arm, Trunk (A) (R) | Linear<br>Trunk, back, limbs (L) |
| Iris heterochromia | | - | - | - | - | - | - | - | - | - | + | - | - | - | - | + |
| Unilateral overgrowth | | Lower limbs | R | - | - | - | - | - | Lower limbs | R | Lower limbs | - | L | - | - | - |
| Macrocephaly | | + | + | + | - | + | + | - | + | + | + | + | - | - | - | + |
| Neurodevelopmental disorder | ID | + | + | ++ | - | - | ++ | - | +++ | + | ++ | +++ | +++ | +++ | + | +++ |
| | ASD | - | - | - | - | - | - | - | + | - | - | - | + | + | - | + |
| | Epilepsy | - | + | + | + | - | - | + | - | - | + | + | + | + | + | + |
| Brain MRI | Phenotype | MEG | HMEG (R) | HMEG (R) | NA | N | N | NA | NA | MEG | HMEG (R) | Mild HMEG (R) | HMEG (L) | NA | HMEG (R) | NA |
| | Asymmetry of LV | - (narrow FH of both LV) | Narrow FH of R LV | Narrow FH of R LV | | - | - | | | - | Narrow FH of R LV | - | Narrow FH of L LV, enlarged L thalami and caudate nuclei | - | | |
| | Corpus callosum | Globally thick | Asymmetry (R thicker + shorter) | Asymmetry (R shorter) | | N | N | | | N | N | N | Asymmetry (L thicker) | Mild asymmetry | | |
| | WM volume | Increased (both hemispheres) | N | Increased (R) | | N | N | | | N | Increased (R) | Increased (R) | Increased (L) | | Increased (R) | |
| | WM signal | N | N | N | | N | N | | | N | N | Bilateral frontal neuronal heterotopias | N | | N | |
| | Cerebral cortex | N | N | N | | N | N | | | N | Thick (R) | N | L thick cortical mantle | N | | |
| | Cerebellar hemispheres | N | Asymmetry (R>L) | N | | N | N | | | N | Asymmetry (R>L) | N | Asymmetry (L>R) | | Asymmetry (R>L) | |
| Psychomotor impairment | | + | +++ | + | - | - | ++ | - | +++ | + | + | ++ | +++ | ++ | ++ | + |
| Distal joint hyperlaxity | | - | - | + | + | - | + | - | - | + | - | + | + | - | - | - |
| Hypertelorism | | + | + | + | + | - | - | - | - | + | - | + | + | + | + | - |
| Downslanting palpebral fissures | | + | - | - | + | + | - | - | + | + | - | - | - | - | + | - |
| Depressed nasal bridge | | + | + | - | - | + | + | - | + | + | - | + | - | - | + | - |
| Scalp hair | | Woolly | Curly | - | - | - | - | - | - | - | - | - | - | Sparse | Woolly | - |
| Anteverted nares | | + | - | - | + | - | - | - | + | + | - | - | + | - | - | - |
| Teeth malposition | | - | - | - | - | - | - | - | - | - | - | + | + | - | - | + |
| High forehead | | - | + | + | + | - | - | + | + | + | + | + | + | + | + | - |
| Frontal bossing | | - | + | - | - | - | - | - | + | + | - | + | - | + | - | - |
| Other features | | Low hair line, microstomia | Thick upper lip, epicanthus | Synophris, low hair line, dysplastic ears | Long philtrum, thin upper lip | Sandal gap | Facial asymetry | - | Telecantus epicanthus | - | - | Thick upper lip, high-arched palate | - | - | - | - |
| Ocular anomalies | | ND | Suspected | S | H | - | - | - | S, H, amblyopia | A, myopia | Coloboma | ND | RP | - | - | Left partial cataract |
| *MTOR* variant | | p.(Lys1452Asn) | p.(Cys1483Tyr) | p.(Ala1519Val) | p.(Ala1519Thr) | p.(Glu1799Lys) | p.(Glu1799Lys) | p.(Thr1977Ile) | p.(Thr1977Ile) | p.(Thr1977Ile) | p.(Ile2017Thr) | p.(Ser2413Ile) | p.(Glu2419Lys) | p.(Glu2419Lys) | p.(Leu2427Pro) | p.(Ile2501Phe) |
| VAF (HI) | | 27% | 10% | 16% | 14% | ND | 27% | 11% | 40% | ND | 10% | 23% | 29% | 21% | 25% | 19% |
| VAF (Blood) | | 15% | - | - | ND | 30% | ND | - | 1% | 4% | 1% | - | - | - | - | - |

* :at last examination, Y: year, ID: intellectual disability, ASD: autistic spectrum disorder; MEG:megalencephaly; HMEG:hemimegalencephaly; WM: white matter; ND: not determined; FH: Frontal horn; R: right-sided; L: left-

sided; A: anterior; P: posterior; +: presence; -: absence. ND : not determined ; S: strabismus; H: hypermetropia; As:astigmatism; RP: Retinitis pigmentosa

**Supplementary Table 5 .** Reports of individuals with hypomelanosis of Ito and hemimegalencephaly

| Report | Linear hypo-pigmentation | Brain MRI findings | Seizures | Psychomotor delay or ID | Body hemi-hypertrophy | Ocular anomalies | Other features |
|---|---|---|---|---|---|---|---|
| (Peserico et al. 1988)[8] | + | HMEG, LV abnormal shape | + | + | - | Divergent strabismus | - |
| (Battistella et al. 1990)[9] | + | HMEG, LV abnormal shape, abnormal periventricular WM signal | + | + | - | Divergent strabismus hypo-pigmented iris | - |
| (Battistella et al. 1990)[9] | + | HMEG, abnormal periventricular WM signal | - | - | - | Divergent strabismus | Facial dysmorphism, dysondotiasis |
| (Williams and Elster 1990)[10] | + | HMEG, abnormal periventricular WM signal | - | + | + | Optic nerve abnormality, retinal detachment, cataract | Imperforate anus, colon atresia, syndactyly clinodactyly, cleft palate, bilateral conductive hearing loss, facial asymmetry, dental anomalies |
| (Malherbe et al. 1993)[11] | + | HMEG, enlarged LV, pachygyria, poor delineation grey-WM | + | + | - | - | - |
| (Tagawa et al. 1994)[12] | + | HMEG, cortical thickening, pachygyria, LV abnormal shape, poor delineation grey-WM | + | - | - | Hypo-pigmented iris | - |
| (Steiner et al. 1996)[13] | + | HMEG, LV abnormal shape, poor delineation grey-WM | + | + | - | - | Hemiparesis |
| (Auriemma et al. 2000)[14] | + | HMEG, cortical thickening, pachygyria, LV abnormal shape | + | Unknown | + | - | Hypotonia |
| (Chapman and Cardenas 2008)[15] | + | HMEG | + | + | - | - | - |
| (Sharma et al. 2009)[16] | + | HMEG, cortical thickening | + | + | + | - | - |
| (Assogba et al. 2010)[17] | + | HMEG, calcifications in caudate and lentiformis nuclei | + | + | + | - | Mild liver enlargement |
| (Lee and al, 2012)[3] | + | HMEG, cortical dysplasia, ectopic and cytomegalic neurons | - | Unknown | Unknown | Unknown | Unknown |
| (Okanari et al. 2014)[18] | + | HMEG, right FCD, enlargement right cerebral hemisphere | + | + | - | Unknown | Hypotonia |
| (Cuddapah et al, 2015)[19] | + | HMEG, unilateral enlargement of left parietal and occipital lobe | + | + | - | - | - |
| (Pelorosso et al. 2019)[7] | + | HMEG, enlarged ventricles, brain midline rightward deviation, cortical thickening, abnormal WM signal | + | + | - | - | - |

HMEG, hemimegalencephaly; MRI, Magnetic resonance imaging; ID, intellectual disability; WM, white matter; LV: Lateral ventricule.; FCD: focal cortical dysplasia.

## SUPPLEMENTARY FIGURES

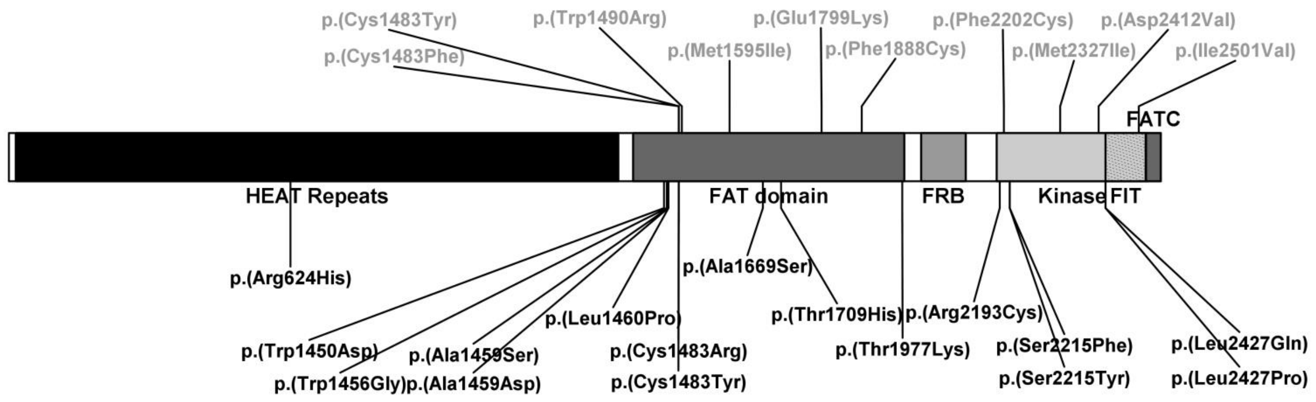


**Supplementary Figure 1. Schematic representation of MTOR protein structure with previously reported germinal (light gray) and somatic variants (black)**[3–7,23–29]**.** FAT: FRAP-ATM-TRRAP domain ; FRB : FKBP12-Rapamycin Binding domain ; FATC: C-terminal FAT domain.

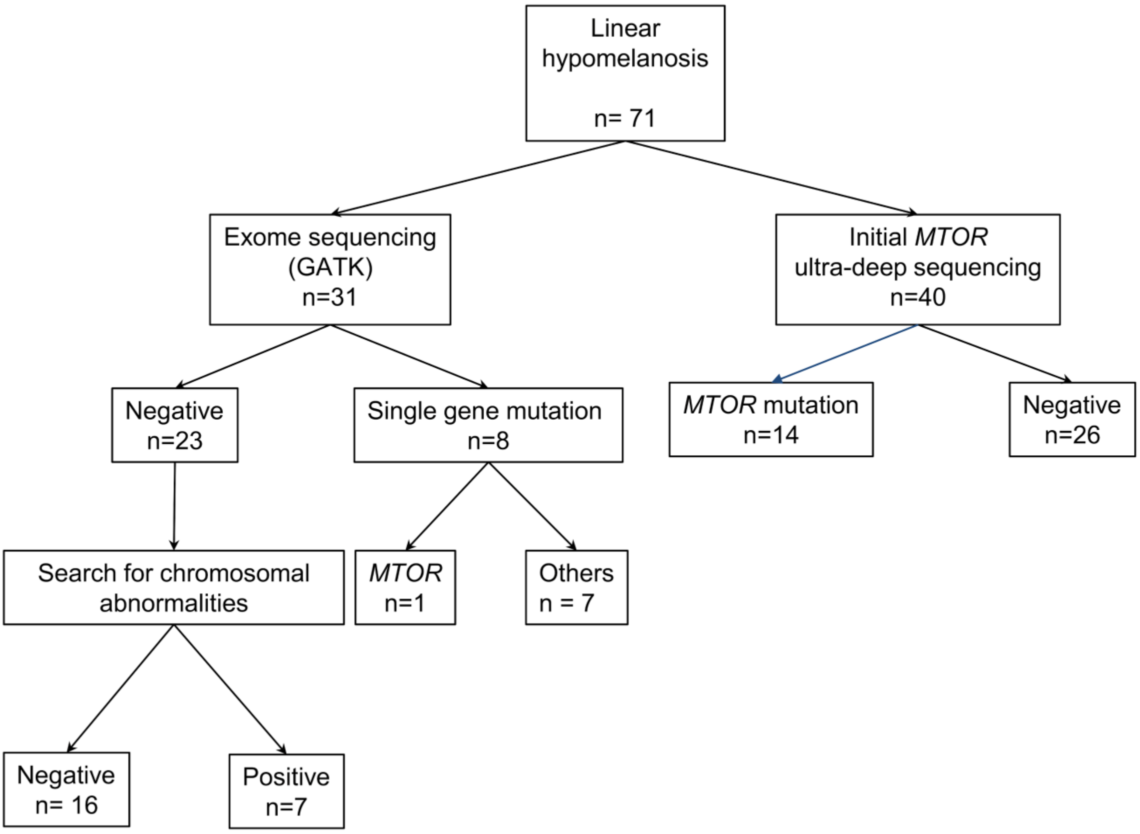


**Supplementary Figure 2.** Next generation sequencing approach in patients with hypomelanosis of Ito.

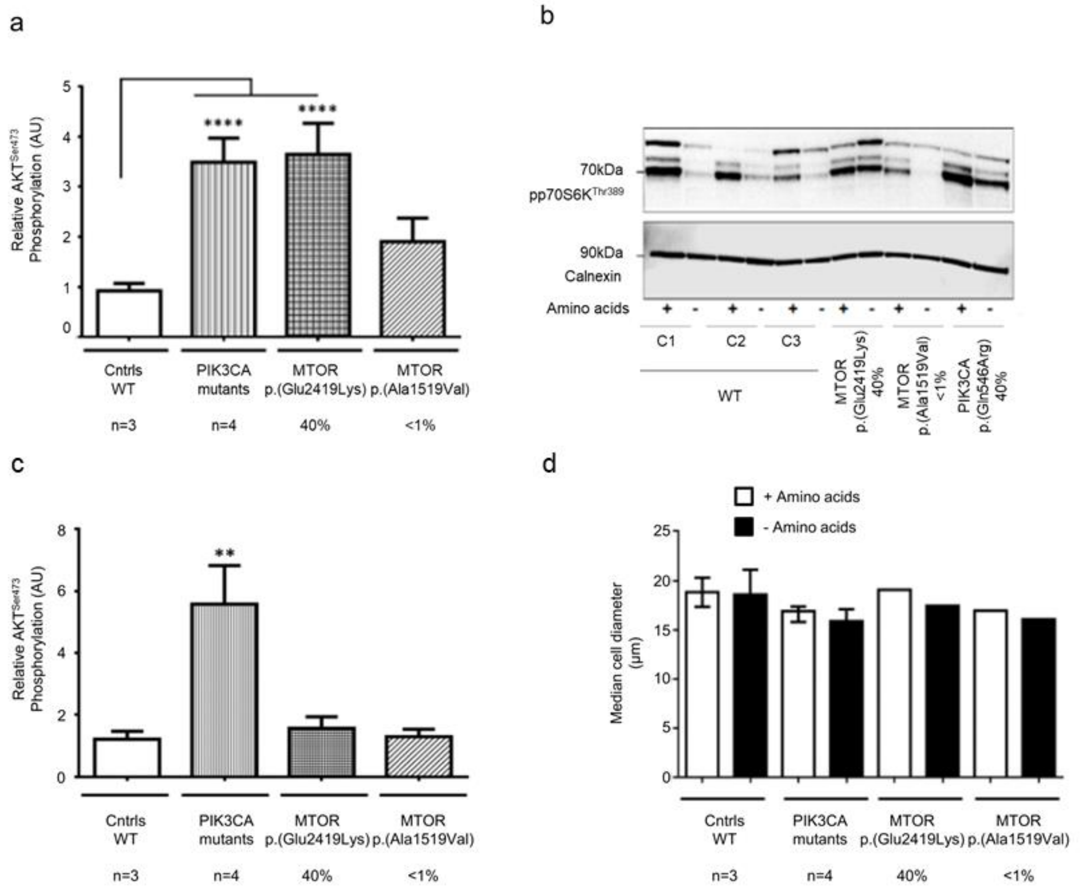


**Supplementary Figure 3. Evaluation of AKT$^{ser473}$ and p70S6K$^{thr389}$ phosphorylation in *MTOR* mutant cell lines following amino acid deprivation (a-b), without amino acid deprivation (c) and cell size in *MTOR* mutant cell lines (d). (a)** ELISA based evaluation of AKT$^{ser473}$ phosphorylation following 50 minutes of amino acid deprivation in wild type control dermal fibroblasts (Cntrls, n=3), *PIK3CA* mutant fibroblast cell lines (n=4 with respective variant allele fractions; M020 [p.(Gly1049Arg) / 40%], M098 [p.(Glu418Lys) / 32%], M018 [p.(Gln546Lys) / 40%] and M032 [p.(His1047Arg) / 30%]) and *MTOR* mutant fibroblast cell lines p.(Glu2419Lys) (VAF = 40%) and p.(Ala1519Lys) (VAF < 1%). Skin fibroblasts from patients with *PIK3CA* variants were used as positive controls. Data is pooled from three independent experiments and error bars represent standard error of the mean (SEM). **** $p < 0.0001$ One-way ANOVA, Tukey's post-hoc analyses. **(b)** Western blot of p70S6K phosphorylation with or without 50 minutes of amino acid deprivation of control wild type cells (C1-C3), a *PIK3CA* mutant cell line (M18, p.(Gln546Lys) 40%) and two

*MTOR* mutant cell lines (p.(Glu2419Lys) and p.(Ala1519Val)). Representative of four independent experiments; calnexin has been used as a loading control. **(c)** ELISA based evaluation of AKT$^{ser473}$ phosphorylation without amino acid deprivation in wild type control cells (Cntrls, n=3), *PIK3CA* mutant cell lines (n=4 with respective pathogenic variation burdens; M020 [p.(Gly1049Arg) 40%], M098 [p.(Glu418Lys) 32%], M018 [p.(Gln546Lys) 40%] and M032 [p.(His1047Arg) 30%]) and *MTOR* mutant cell lines p.(Glu2419Lys) (40% pathogenic variation burden) and p.(Ala1519Lys) (<1% pathogenic variation burden). Data is pooled from three independent experiments and error bars represent SEM. **p < 0.01 One-way ANOVA, Tukey's post-hoc analyses. **(d)** Median cell diameter using a FACS-based multi-sizer with or without amino acid deprivation for 50 minutes. Pathogenic variation burdens are indicated below. Error bars represent SEM; 10,000 cells were counted in total. Skin fibroblasts from patients with known activating *PIK3CA* variants were used as positive controls and cultured primary fibroblasts carrying p.(Ala1519Val) pathogenic variation were used for comparison.

## Supplementary bibliography


1. Sorlin A, Maruani A, Aubriot-Lorton M-H, et al. Mosaicism for a KITLG Mutation in Linear and Whorled Nevoid Hypermelanosis. *Journal of Investigative Dermatology*. 2017;137(7):1575-1578.

2. DePristo MA, Banks E, Poplin RE, et al. A framework for variation discovery and genotyping using next-generation DNA sequencing data. *Nat Genet*. 2011;43(5):491-498.

3. Lee JH, Huynh M, Silhavy JL, et al. De novo somatic mutations in components of the PI3K-AKT3-mTOR pathway cause hemimegalencephaly. *Nat Genet*. 2012;44(8):941-945.

4. Gordo G, Tenorio J, Arias P, et al. mTOR mutations in Smith-Kingsmore syndrome: Four additional patients and a review. *Clinical Genetics*. 2018;93(4):762-775.

5. Mirzaa GM, Campbell CD, Solovieff N, et al. Wide spectrum of developmental brain disorders from megalencephaly to focal cortical dysplasia and pigmentary mosaicism caused by mutations of MTOR. *JAMA Neurol*. 2016;73(7):836-845.

6. Handoko M, Emrick LT, Rosenfeld JA, et al. Recurrent mosaic MTOR c.5930C > T (p.Thr1977Ile) variant causing megalencephaly, asymmetric polymicrogyria, and cutaneous pigmentary mosaicism: Case report and review of the literature. *Am J Med Genet A*. 2019;179(3):475-479.

7. Pelorosso C, Watrin F, Conti V, et al. Somatic double-hit in MTOR and RPS6 in hemimegalencephaly with intractable epilepsy. *Hum Mol Genet*. Published online August 14, 2019.

8. Peserico A, Battistella PA, Bertoli P, Drigo P. Unilateral Hypomelanosis of Ito with Hemimegalencephaly. *Acta Paediatrica*. 1988;77(3):446-447.

9. Battistella PA, Peserico A, Bertoli P, Drigo P, Laverda AM, Casara GL. Hypomelanosis of Ito and hemimegalencephaly. *Childs Nerv Syst*. 1990;6(7):421-423.

10. Williams DW, Elster AD. Cranial MR imaging in hypomelanosis of Ito. *J Comput Assist Tomogr*. 1990;14(6):981-983.

11. Malherbe V, Pariente D, Tardieu M, et al. Central nervous system lesions in hypomelanosis of Ito: an MRI and pathological study. *J Neurol*. 1993;240(5):302-304.

12. Tagawa T, Otani K, Futagi Y, et al. [Hypomelanosis of Ito associated with hemimegalencephaly]. *No To Hattatsu*. 1994;26(6):518-521.

13. Steiner J, Adamsbaum C, Desguerres I, et al. Hypomelanosis of Ito and brain abnormalities: MRI findings and literature review. *Pediatr Radiol*. 1996;26(11):763-768.

14. Auriemma A, Agostinis C, Bianchi P, et al. Hemimegalencephaly in hypomelanosis of Ito: early sonographic pattern and peculiar MR findings in a newborn. *European Journal of Ultrasound*. 2000;12(1):61-67. d

15. Chapman K, Cardenas JF. Hemimegalencephaly in a Patient With a Neurocutaneous Syndrome. *Seminars in Pediatric Neurology*. 2008;15(4):190-193.

16. Sharma S, Sankhyan N, Kabra M, Kumar A. Hypomelanosis of Ito with hemimegalencephaly. *Dermatol Online J*. 2009;15(11):12.

17. Assogba K, Ferlazzo E, Striano P, et al. Heterogeneous seizure manifestations in Hypomelanosis of Ito: report of four new cases and review of the literature. *Neurological Sciences*. 2010;31(1):9-16.

18. Okanari K, Miyahara H, Itoh M, et al. Hemimegalencephaly in a Patient With Coexisting Trisomy 21 and Hypomelanosis of Ito. *J Child Neurol*. 2014;29(3):415-420.

19. Cuddapah VA, Thompson M, Blount J, Li R, Guleria S, Goyal M. Hemispherectomy for Hemimegalencephaly Due to Tuberous Sclerosis and a Review of the Literature. *Pediatric Neurology*. 2015;53(5):452-455.

20. Cooper GM, Stone EA, Asimenos G, Green ED, Batzoglou S, Sidow A. Distribution and intensity of constraint in mammalian genomic sequence. *Genome Res*. 2005;15(7):901-913.

21. Adzhubei IA, Schmidt S, Peshkin L, et al. A method and server for predicting damaging missense mutations. *Nat Methods*. 2010;7(4):248-249.

22. Kircher M, Witten DM, Jain P, O'Roak BJ, Cooper GM, Shendure J. A general framework for estimating the relative pathogenicity of human genetic variants. *Nat Genet*. 2014;46(3):310-315.

23. EPI4K Consortium, Epilepsy Phenome/Genome project. De novo mutations in the classic epileptic encephalopathies. *Nature*. 2013;501(7466):217-221.

24. Smith L, Saunders CJ, Dinwiddie DL, et al. Exome Sequencing Reveals De Novo Germline Mutation of the Mammalian Target of Rapamycin (MTOR) in a Patient with Megalencephaly and Intractable Seizures. *Journal of Genomes and Exomes*. 2013;2:63-72.

25. Baynam G, Overkov A, Davis M, et al. A germline MTOR mutation in Aboriginal Australian siblings with intellectual disability, dysmorphism, macrocephaly, and small thoraces. *American Journal of Medical Genetics Part A*. 2015;167(7):1659-1667.

26. D'Gama AM, Geng Y, Couto JA, et al. mTOR Pathway Mutations Cause Hemimegalencephaly and Focal Cortical Dysplasia. *Ann Neurol*. 2015;77(4):720-725.

27. Lim JS, Kim W, Kang H-C, et al. Brain somatic mutations in MTOR cause focal cortical dysplasia type II leading to intractable epilepsy. *Nature Medicine*. 2015;21(4):395-400.

28. Moosa S, Böhrer-Rabel H, Altmüller J, et al. Smith–Kingsmore syndrome: A third family with the MTOR mutation c.5395G>A p.(Glu1799Lys) and evidence for paternal gonadal mosaicism. *American Journal of Medical Genetics Part A*. 2017;173(1):264-267.

29. Rodríguez-García ME, Cotrina-Vinagre FJ, Bellusci M, et al. A novel de novo MTOR gain-of-function variant in a patient with Smith-Kingsmore syndrome and Antiphospholipid syndrome. *Eur J Hum Genet*. 2019;27(9):1369-1378.
